# Supplementary figures and images for: The malaria parasite PP1 phosphatase controls the initiation of the egress pathway of asexual blood-stages by regulating the rounding-up of the vacuole
Source: PLoS Pathog. 2025 Jan 14;21(1):e1012455. doi: 10.1371/journal.ppat.1012455 (PMC11731718; doi:10.1371/journal.ppat.1012455)

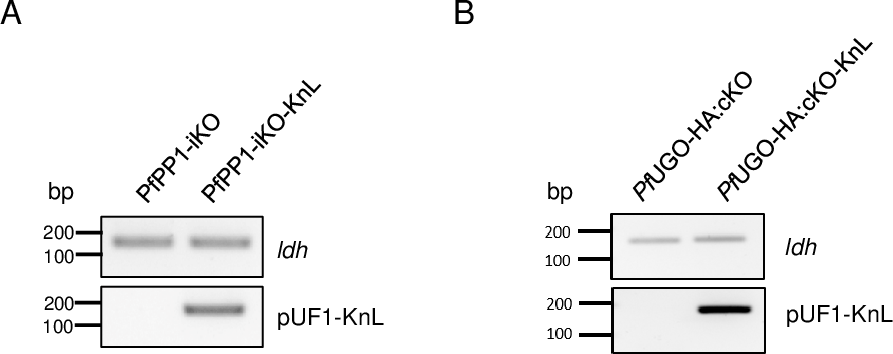

Supplement: S1 Fig — PCR verification of the presence of the KnL reporter in PfPP1-iKO-KnL (A) and PfUGO-HA:cKO-KnL (B) parasite lines, as compared to the parental background. The gene encoding the lactate dehydrogenase (ldh) was used as a control. bp: base pairs. (TIF) [file ppat.1012455.s001.tif]

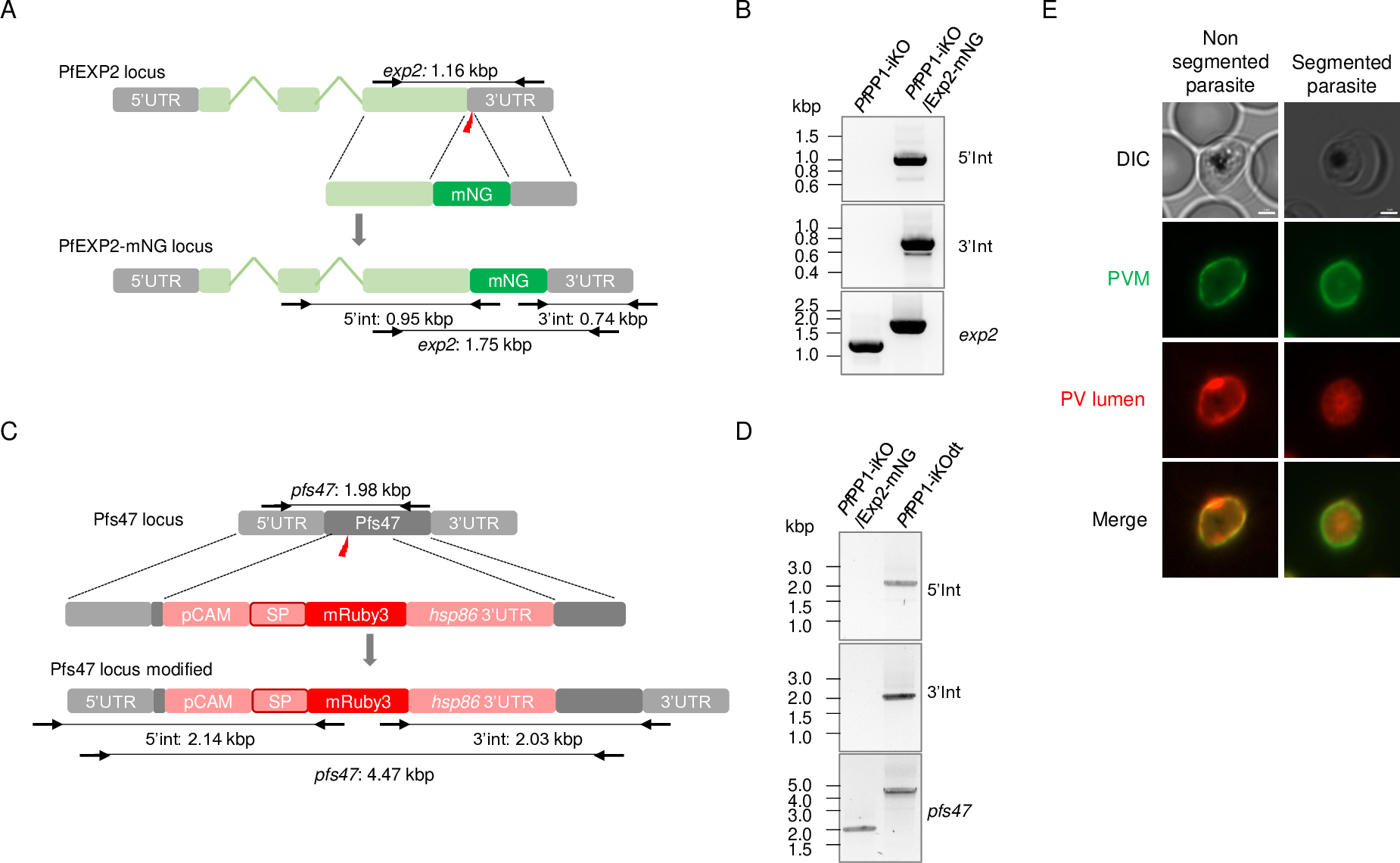

Supplement: S2 Fig — A. Schematic of the C-terminal tagging of exp2 with mNG by CRISPR-Cas9. Green rectangles and lines represent exons and introns, respectively. Dashed lines show the double homologous recombination taking place in the exp2 locus. Red lightning stands for Cas9 double strand break. Integrative PCRs as in B are shown on the edited locus. B. PCR genotyping of PfPP1-iKO/Exp2-mNG parasites, as compared to the parental line. kbp: kilo base pairs. C. Schematic of the integration of mRuby3 in the Pfs47 locus of PfPP1-iKO/Exp2-mNG. Dashed lines show the double homologous recombination taking place at the Pfs47 locus. Red lightning stands for Cas9 double strand break. Integrative PCRs as in D are shown on the edited locus. D. PCR genotyping of PfPP1-iKOdt parasites, as compared to the parental line. Amplification of the Pfs47 locus shows the extension of the locus due to mRuby3 construct integration in the edited parasites. kbp: kilo base pairs. E. Validation of PfPP1-iKOdt parasite line by live-microscopy. Scale bar = 2 μm. (TIF) [file ppat.1012455.s002.tif]

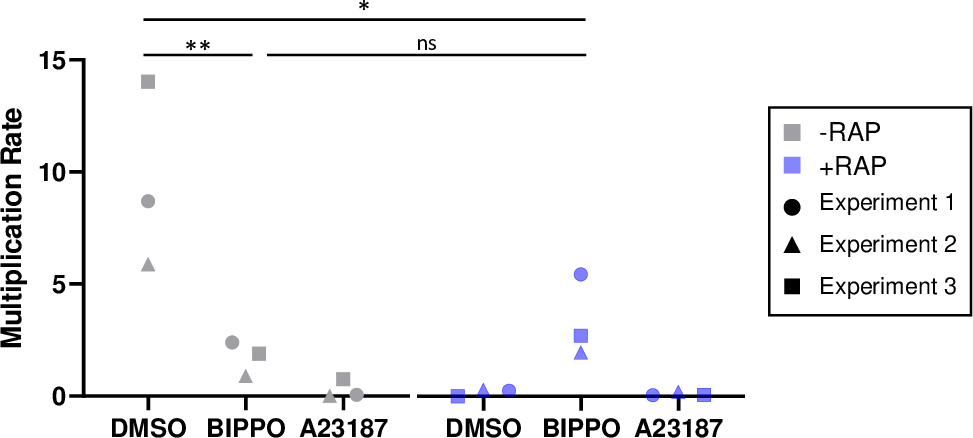

Supplement: S3 Fig — Quantification of invasion on Giemsa-stained blood smears. The multiplication rate upon egress of PfPP1-iKOdt parasites treated ± RAP was quantified following 1 h of pharmacological treatment by microscopic examination of thin blood smears and counting of schizonts and ring stages (n = 3 biological replicates). Statistical analyses were performed by one way Anova with Tukey’s multiple comparisons test, with ** p ≤ 0.005; * p ≤ 0.05; ns = non-significant. (TIF) [file ppat.1012455.s003.tif]

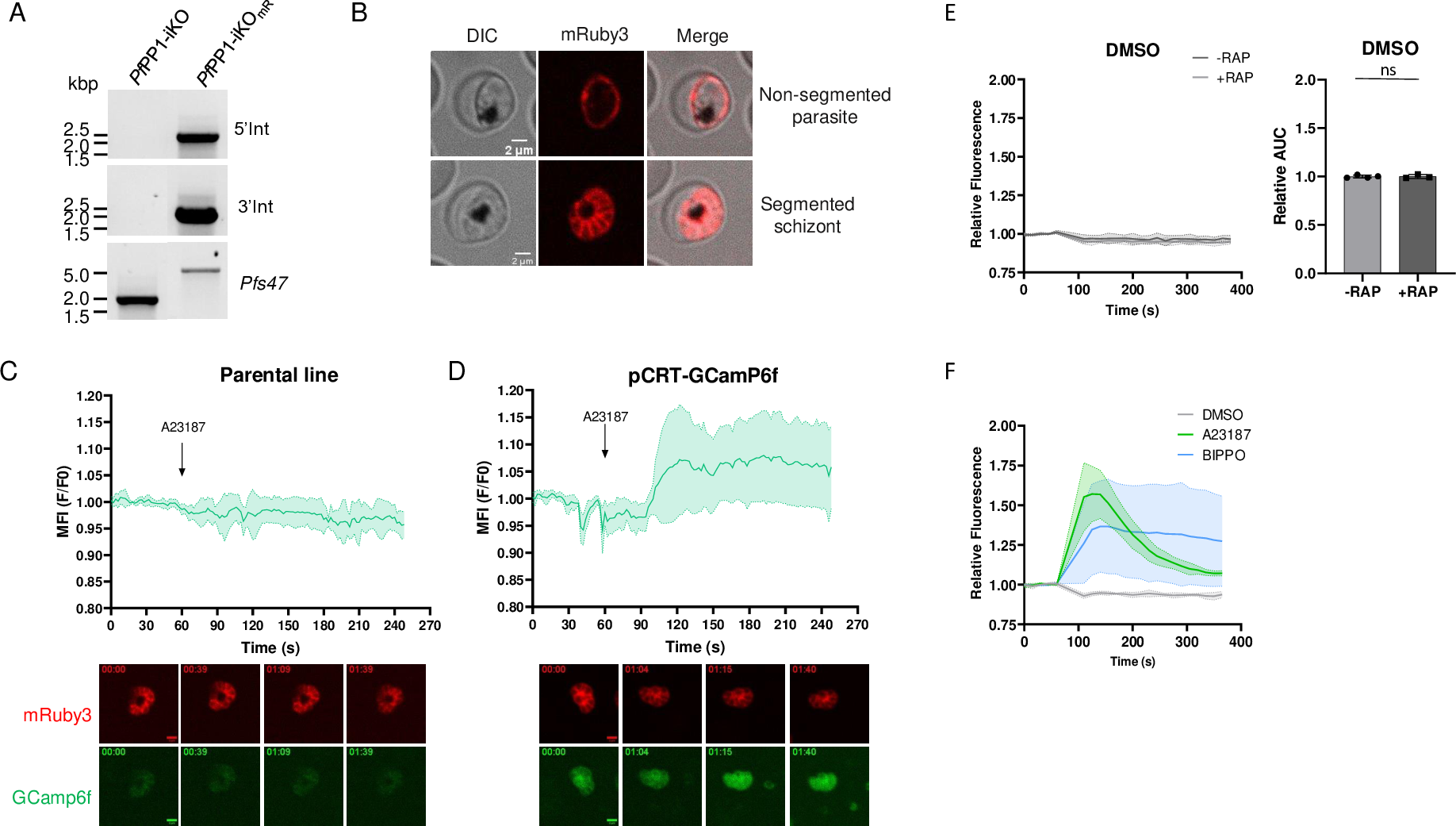

Supplement: S4 Fig — A. PCR genotyping of PfPP1-iKOmR parasites, as compared to the parental line. B. Still frames of PfPP1-iKOmR parasites from live microscopy. Scale bar = 2 μm. C-D. Top: Mean fluorescence intensities (MFI ± SD) of 5 representative parasites from PfPP1-iKOmR line (C), or PfPP1-iKOmR-pCRT-GCamp6f line (D). MFI was normalized to the fluorescence baseline recorded prior to the addition of A23187. Bottom: Still frames from live-microscopy experiment performed on the same parasites as depicted above. E. Determination of Ca2+ levels in PfPP1-iKOmR/pCRT-GCamp6f strain in a saline buffer containing Ca2+, upon treatment with vehicle. Left: the fluorescence of GCamp6f was normalized to the baseline prior to the addition of DMSO. Right: Comparison of the areas under the curve (AUC). Statistical analyses were performed by unpaired t-test with ** p ≤ 0.01; ns = non-significant (n = -5 independent experiments). F. Determination of Ca2+ levels in PfPP1-iKOmR/pCRT-GCamp6f strain in a saline buffer deprived of Ca2+. The fluorescence of GCamp6f was normalized to the baseline prior to the addition of the tested compound. n = 3 or 2 independent experiments for A23187 and BIPPO, respectively. (TIF) [file ppat.1012455.s004.tif]
