# Supplementary material for: The malaria parasite PP1 phosphatase controls the initiation of the egress pathway of asexual blood-stages by regulating the rounding-up of the vacuole
Source: PLoS Pathog. 2025 Jan 14;21(1):e1012455. doi: 10.1371/journal.ppat.1012455 (PMC11731718; doi:10.1371/journal.ppat.1012455)
Supplement: Attachment — Submitted filename: Response-To-Reviewers.pdf [file ppat.1012455.s012.pdf]

We thank the reviewers for their thoughtful comments and suggestions, which we believe helped improve the manuscript. Please, see below our response to each comment and issue raised by the reviewers.

Reviewer #1

1. Fig 1A could either include clearer labelling in the figure or describe what the cells represent more thoroughly in the figure legend

We made revisions of the figure legend to improve the clarity of the schematic (lines 877-879) and added some labelling in the figure.

2. Fig 1B – the authors could say how many cells were measured for each condition

We have used 4.10^4 parasites per condition. It is now stated in the Materials & Methods section, line 554.

3. Fig 1C – the authors should describe the constructs in this figure legend in the same order as they appear in the figure – the order of the descriptions in the legend does not currently match the order of constructs in the schematic

The order of the constructs has been modified in the legend to match the one of the schematic (lines 889-893).

4. Fig 1D – the authors could describe what the numbers mean in the top left corner of each image (presumably time – include unit)

The numbers in the top left corner are now described in the figure legend (lines 894-895).

5. Fig 1E – I'm not sure if unpaired t test is the most appropriate statistical test when comparing so many groups. One way ANOVA with post hoc testing might have been a better choice

The statistical analysis has been re-done with a one-way ANOVA, as suggested by the reviewer.

6. Fig 2A – the authors could include fluorescence markers in the labelling on image panels – e.g. PVM-neon green. Indicate a unit for time in the figure legend

We have revised the figure and the legend (lines 908-909) according to the reviewer suggestions.

7. Fig 2B-D – more information could be given about exactly how these times are measured – how do they decide when to start measuring? E.g. T1 is a measure of the whole egress process whereas T3 is a measure of just the rupture process. When do you start to measure rupture alone? Is this open to interpretation?

We have revised the manuscript to clarify how and when the different times were measured (lines 206-215).

It is also not clear why the authors mention t4 and T5 if they did not give any data on it in this figure. It was given in figure 5 C and D. Can they be either pooled or the T4 and T5 removed from Fig 2 as it leads to confusion.

We have removed T4 and T5 from Fig. 2A, as suggested by the reviewer.

8. Fig 3A – the authors could say how many cells were measured

We used $1{,}25.10^8$ parasites per condition. It is now stated lines 252-253, and can also be found in the Materials & Methods section line 600.

9. Fig 3A-D – it might be useful to include a label on these graphs showing which parasites these experiments were performed in

We have now specified which parasite line has been used for each graph, as suggested by the reviewer.

10. In Fig3C, can authors explain that this evidently clear that inhibition by BIPPO has not changed any Ca++ levels in +RAP parasites. It is almost basal. Then how BIPPO dependent Ca++ enrichment is related to any of the results.

From Fig. 3C, we agree with the reviewer that BIPPO treatment does not lead to any detectable change in cytoplasmic Ca2+ levels in +RAP parasites. This result correlates with lower cGMP levels measured in those same parasites (Fig. 3A), presumably leading to a poor activation of PKG. However, these measurements are performed on the parasite population and not at the single cell level. The fact that BIPPO still induces about 50% of egress of +RAP parasites (Fig. 1E) likely reflects some heterogeneity in the population, with cGMP and Ca2+ levels reaching sufficient levels to trigger egress in some parasites. Therefore, the fact that we do not detect any Ca2+ rise in +RAP parasites in Fig. 3C reflects a strong impairment in PKG-dependent Ca2+ mobilization, but not a proof of its absence, as we may be technically limited in the sensitivity of the reporter, and/or in the signal detection. This point is now discussed in the main text, lines 275-282.

11. Fig 3D – the figure legend says the parasites were treated with A23187 and data normalised to this but this data isn't on the graph – could it be included for comparison?

The luminescence measured upon A23187 treatment was considered as 100%, and used to normalize the values obtained upon treatment with other molecules. Therefore, the data of A23187 would all correspond to 100% and are not shown on the graph.

12. Fig 4A – it would be useful to change the colour of the black arrowhead to something which will stand out more

We have made the suggested modification in Fig. 4B, and the colour of the arrowhead is now red. To answer to a point raised by reviewer's 2, the images of Fig. 4B have also been changed.

13. Fig 4C – the data here is only from a single experiment. Could another repeat be added?

We have performed a second TEM experiment, and the graph in Fig. 4C now represents two biological replicates.

14. Fig 5A – the authors could include a unit for time for the still images in the legend or figure itself.

The numbers in the top left corner of each frame are now described in the figure legend (lines 948-949).

15. Fig5C and D can be either pooled to fig 2 or the fig2 and 5 can be put together. The schematic for the different time in Fig2 should be given in Fig5 again so that its clear what t4 or T5 represent.

According to the reviewer's suggestion, we have added in Fig. 5C the same schematic as in Fig. 2A, with an annotation for T4 and T5.

16. Line 220: How the measurement of cGMP was performed

cGMP measurement was performed by ELISA. It is now stated line 251, and can also be found in the Material & Methods section, line 599.

17. Line 224/228: mention fig 3A
The revision has been made, lines 255 and 259.

18. Line 232: schematic and fluorescent images of cytosolic ca++ can be detailed in supplementary.
Fluorescent images of GCamp6f are already provided in S6 appendix.

Reviewer #2

1. Throughout the manuscript, some percentages and numbers are provide without standard deviation (e.g., line 161, line 163, line 297, line 299). All measurements should have XXX +/- XXX to give the reader some insight about the variability of the measurement.

We agree with the reviewer's assessment and we now provide numbers with standard deviation (lines 137, 144, 162-166, 335-337, 348).

2. How is the initial time point determined to begin measuring T1, T2, and T4 determined? Is this when the imaging is started or is there is a specific characteristic that defines this time point?
We have revised the manuscript to clarify how and when the different times were measured (lines 206-215).

3. In the study, the authors have attempted to calculate the leakage of mRuby3 signal to the host cytoplasm upon PV rupture. However, given the resolution power of the method used, such calculations seem a little precarious.

We agree with the reviewer that the method is not highly resolutive. However, it has been used by Hale et al. to evidence PVM permeabilization. The authors report the leakage of a fluorescent mCherry PV reporter into the host cytosol prior to rounding-up. As our mRuby3 reporter is of similar molecular weight, we expected it to leak into the host cell via the same pathway.

4. Fig 4: The parasites are treated with C2 which would be expected to block PKG activity and, thus, block PVM poration. Did the authors wash out the C2 before the TEM preparation? The parasites for TEM were treated at 41 hpi and collected at 44 hpi for fixation (lines 569-572), which is likely a few hours before egress. Having said that, the results of electron microscopy showing the absence of PV poration could be considered if the contrast measurement was objective and details were provided. It is currently unclear as to how was the presence or absence of equalization in Figure 4B-C measured.

In our culture conditions, the erythrocytic cycle takes ~ 44h, which is why we treated the parasites with C2 at 41 hpi and collected them at 44 hpi. We have clarified the length of the erythrocytic cycle in the main text (line 131-132), as this is a crucial information pertinent to the timing of egress, as pointed by the reviewer. We did not wash out C2 before TEM preparation, as PVM poration would be PKG-independent, as stated in Hale et al., and further confirmed by the corresponding author upon request.

As for contrast measurement, we strongly believe that TEM is primarily a qualitative method, that can be quantitative in terms of number of events, but not necessarily in terms of pixel quantification. Indeed, the mean grey values depend on many factors, inherent to red blood cell

age, sample preparation, section thickness, beam brightness…. Therefore, we focused our analysis on segmented parasites, that displayed an apparent and intact surrounding PVM as shown in Fig. 4B. PVM was considered as “porated” when no difference of contrast could be detected by eye between the PV lumen and the RBC cytoplasm. For each set of images, two independent analysis were performed by two researchers. We are providing new images in Fig.4B for further clarity and tempered our statement in the main text, lines 324-326.

5. Do the egress inducer-treated parasites reinvade? Can a productive infection be maintained by sequential treatment with BIPPO or A23187 in the PP1 null parasites?

To answer this question, we have performed invasion assays. A23187 does not lead to any productive invasion, irrelevant of RAP treatment. In control parasites (-RAP), Bippo induces premature egress, resulting in lower invasion rate as compared to the condition where natural egress takes place. However, because of the accumulation of stalled schizonts in +RAP condition, the invasion rate is higher in +RAP parasites than in -RAP parasites. The results are now described in the main text lines 173-191 and shown in S4 Appendix.

6. This is likely something that would be fixed in future iterations, but the locations of the supplemental figures were difficult to determine.

We have modified the references to the supplemental figures to improve their locations and identifications.

7. The references should be checked for language. I think the dates are in French in some references.
The language of the references has been checked and modified accordingly.

8. Figures labelled as DIC seem to be brightfield images and might be worth checking and correcting.
After verification, we confirm that the images correspond to DIC.

9. The restoration of PV rounding up in A23187 parasites is also partial (~50%) albeit higher than that of BIPPO (~30%) as per figure 5B. The statement in lines 314 and 315 needs to be edited accordingly.

We have edited the main text, according to the reviewer’s suggestion, line 354-355.

Reviewer #3

1. Were any PCRs etc, done to show the loxP flanked PP1 was being deleted efficiently? Given that parasites did not egress Fig 1B, one would assume the deletion was efficient.
The reviewer’s assumption is correct. The verifications that *pp1* gene was correctly excised and led to PP1 protein depletion upon RAP treatment were published in Paul et al. 2020. In addition, the fact that RAP-treated parasites do not egress is a proof of the efficient gene excision.

2. In Fig 2, the length of time each egress stage lasts are measured. I may have missed this, but how was it determined when to start the measuring? Was this simply done by synchronising the parasites and observing from 43 hpi for example?
We have revised the manuscript to clarify how and when the different times were measured (lines 206-215).

3. Line 194 – 195. What is meant by “egress molecules primarily modulate PVM breaching”?
We have rephrased this sentence in “egress molecules primarily modulate the kinetics of PVM breaching” to improve clarity (line 224-225).

4. Line 200+ . “correlated with an acceleration of T2 (T2: 468 ± 21 sec, ~8 min)”What is being compared to what here because the last two A23187 columns in 2C are NS.
Here, the acceleration of T2 was compared to natural egress. It is now specified in the text (line 233).

5. A citation to Fig 2D appears to be missing.
We have added citations to Fig.2D. It can now be found lines 217, 223, 239.

6. In Fig 5SC Top, is the red MFI trace referring to mRuby3 or to GCamp6f? This refers to parts D and E too.

We agree that the colours used for the trace might have been misleading. All the MFI of Fig. S5 (now Fig. S6) correspond to the fluorescence of GCamp6f. The trace of Fig. S5C was changed in green, as in S5D. However, in S5E and S5F, we kept different colours for traces as each one corresponds to the fluorescence measured upon stimulation with a given inducer, as in Fig.3B-C: DMSO in grey, A23187 in green and BIPPO in blue.

7. Line 314. “Importantly, the defect in rounding-up of PfPP1-null parasites (+RAP) was overcome by A23187, while BIPPO only provided a partial complementation (Fig. 5B).” How can A23187 overcome the +Rap effect when it is NS compared to -Rap?
PfPP1-null parasites (+ RAP) do not experience the rounding-up step (Fig. 5A). However, A23187 treatment restores rounding-up. As the observed % is not significantly different from the one observed in control parasites (-RAP) (NS), it means that the calcium ionophore overcomes the +RAP effect.

8. In Fig 5A, are the pie charts % or the actual number of cells counted?
The numbers in the pie charts represent % and the figure has been modified to clarify it. In the figure legend, the number of parasites analysed is given for each condition (lines 951-952).
